# Analyses of African common bean (*Phaseolus vulgaris* L.) germplasm using a SNP fingerprinting platform: diversity, quality control and molecular breeding

**DOI:** 10.1007/s10722-019-00746-0

**Published:** 2019-02-19

**Authors:** Bodo Raatz, Clare Mukankusi, Juan David Lobaton, Alan Male, Virginia Chisale, Berhanu Amsalu, Deidré Fourie, Floride Mukamuhirwa, Kennedy Muimui, Bruce Mutari, Susan Nchimbi-Msolla, Stanley Nkalubo, Kidane Tumsa, Rowland Chirwa, Mywish K. Maredia, Chunlin He

**Affiliations:** 1 Present Address: Bean Program, International Center for Tropical Agriculture (CIAT), A.A. 6713, Cali, Colombia; 2 International Centre for Tropical Agriculture (CIAT), 15 km Bombo Road, Kawanda, Box 6247, Kampala, Uganda; 3 School of Environmental and Rural Sciences, University of New England, Armidale, Australia; 4 Chitedze Research Station, Department of Agricultural Research Services (DARS), P.O. Box 158, Lilongwe, Malawi; 5 Melkassa Agricultural Research Center, Ethiopian Institute of Agricultural Research (EIAR), P.O. Box 436, Adama, Ethiopia; 6 ARC-Grain Crops Institute, Private Bag X1251, Potchefstroom, 2520 South Africa; 7 Rwanda Agriculture Board (RAB), P.O. Box 5016, Kigali, Rwanda; 8 Misamfu Regional Research Station, Zambia Agriculture Research Institute (ZARI), Box 410055, Kasama, Zambia; 9 Crop Breeding Institute, Department of Research and Specialist Services (DR&SS), Harare, Zimbabwe; 10 Sokoine University of Agriculture, Morogoro, Tanzania; 11 National Crops Resources Research Institute (NaCRRI)-Namulonge, P. O. Box 7084, Kampala, Uganda; 12 International Centre for Tropical Agriculture (CIAT), Mchinji Road, P.O. Box 158, Lilongwe, Malawi; 13 Michigan State University, East Lansing, USA; 14 Genotyping Support Services (GSS), Generation Challenge Programme (GCP), c/o CIMMYT, Mexico, Mexico; 15 Present Address: Noble Research Institute, LLC, Ardmore, OK USA

**Keywords:** DNA fingerprinting, SNP genotyping, Diversity analysis, Germplasm purity, Marker assisted selection

## Abstract

Common bean (*Phaseolus vulgaris* L.) is an important staple crop for smallholder farmers, particularly in Eastern and Southern Africa. To support common bean breeding and seed dissemination, a high throughput SNP genotyping platform with 1500 established SNP assays has been developed at a genotyping service provider which allows breeders without their own genotyping infrastructure to outsource such service. A set of 708 genotypes mainly composed of germplasm from African breeders and CIAT breeding program were assembled and genotyped with over 800 SNPs. Diversity analysis revealed that both Mesoamerican and Andean gene pools are in use, with an emphasis on large seeded Andean genotypes, which represents the known regional preferences. The analysis of genetic similarities among germplasm entries revealed duplicated lines with different names as well as distinct SNP patterns in identically named samples. Overall, a worrying number of inconsistencies was identified in this data set of very diverse origins. This exemplifies the necessity to develop and use a cost-effective fingerprinting platform to ensure germplasm purity for research, sharing and seed dissemination. The genetic data also allows to visualize introgressions, to identify heterozygous regions to evaluate hybridization success and to employ marker-assisted selection. This study presents a new resource for the common bean community, a SNP genotyping platform, a large SNP data set and a number of applications on how to utilize this information to improve the efficiency and quality of seed handling activities, breeding, and seed dissemination through molecular tools.

**Electronic supplementary material:**

The online version of this article (10.1007/s10722-019-00746-0) contains supplementary material, which is available to authorized users.

## Introduction

The common bean (*Phaseolus vulgaris* L.) also known as dry bean is the most important food legume crop for direct human consumption grown worldwide (Broughton et al. 2003). It also serves as a source of income for smallholder farmers and as a source of foreign exchange earnings through export in some African countries like Ethiopia, where bean exports are valued at more than 100 million USD per annum (Amsalu et al. 2016). The crop is adaptable to many different cropping systems and has a short growing cycle making it attractive to many farmers in different regions of the world. The bean crop is also a good source of protein, calories and micronutrients. Due to its high nutritional value, it is particularly important for poor smallholder farmers in low input agriculture systems.

Genetic diversity in common bean germplasm can be categorised into two major genepools: (1) the Andean genepool (large seeded) comprised of the races Chile, Nueva Granada and Peru; and (2) the Mesoamerican genepool comprised of the races Jalisco, Durango, Guatemala, and Mesoamerica (all small seeded) (Singh et al. 1991a). The classification of genepools has been repeatedly reported based on the relationship between seed size (small versus large) and the *Dl* genes (*Dl*-*1* versus *Dl*-*2*) (Shii et al. 1980), through F1 hybrid incompatibility (Gepts and Bliss 1985), phaseolin seed proteins (Gepts et al. 1986), allozymes (Singh et al. 1991b), morphological traits (Singh et al. 1991c) and DNA markers (Khairallah et al. 1990).

Various molecular marker systems have been used to study common bean diversity at the molecular level; RFLP markers confirmed the division into two gene pools (Velasquez and Gepts 1994). AFLP makers were used in the discovery that Andean genepool had a narrow genetic base (Beebe et al. 2001). Most recently, Cichy et al. (2015a) characterized an Andean diversity panel (ADP) using the Illumina BARCBean6K_3 SNP chip and showed differentiation in distinct groups based on origin and grain type. Whole genome sequencing data were also used to evaluate intra and inter specific diversity in 12 *Phaseolus* species establishing the relatedness of common bean sister species (Rendón-Anaya et al. 2017; Lobaton et al. 2018).

A wide germplasm diversity is a valuable resource for breeding programs to tackle a variety of traits. However, utilizing this available diversity in commonly grown bean varieties/landraces as parents in a breeding program faces several challenges for bean breeding programs in Africa. The large diversity of beans grown in the region is not properly characterized and differentiated and often varieties are given different names depending on where they are grown and yet some of them may in fact be genetically identical. It is also a very common practice that bean is grown as a mixture of varieties, which often leads to the loss of identity for some of the varieties. At present, in Uganda many landraces or varieties are not referred to by their names but by their seed size and color e.g., large yellow, large white, or large coffee (dark brown) (Okii et al. 2014). For instance the two most common red mottled bean varieties in Uganda, K132 and NABE4 are given multiple names such as Nambale, Kawomera, Africa, 2000 etc., depending on where they are grown and how they were introduced into the community. Newly released varieties of a similar market class are often given the same name as older varieties whose seed appears similar. This creates a problem in accurately assessing adoption of improved varieties. A more pertinent problem arises when breeders seek for breeding parents from the so called “landraces” as sources of important traits, which further affects the generation, tracking and comparisons of breeding lines.

For a breeding program to be successful, it is crucial to have the prior knowledge of the parents about their origin and the characteristics of the important traits. Cultivated landraces of common bean from the primary centers of domestication in Latin America showed specific associations for morphological traits (Singh et al. 1991c; Singh 1989), molecular markers (Khairallah et al. 1990), breeding behaviour (Singh et al. 1993) and geographical and ecological adaptation (Singh 1989). Molecular markers may prove valuable in supporting common bean germplasm development through fingerprinting and characterization of the genetic diversity. This requires an easy to use genotyping method and an established data set for genotypic comparisons.

A number of marker types have been used in the past for several kinds of genetic studies, such as RAPD, RFLP, SSR, cpDNA, AFLP to name a few (Miklas et al. 2006). Discovery and application of these marker systems is a difficult and time-consuming exercise. Single Nucleotide Polymorphism (SNP) markers have demonstrated their utility in genetic studies (Thomson 2014). SNPs are differences in DNA sequence of just one nucleotide and usually bi-allelic. They are the most common type of polymorphism, and are distributed throughout the genome. SNP genotyping can be relatively simple (amendable to automated high throughput platforms), but SNP discovery generally requires extensive DNA sequencing, which has become available through next generation sequencing (NGS) technologies. SNP markers are useful for genetic studies because they are available in large numbers, co-dominant and transferable between different genotypes.

SNP genotyping platforms have been generated in recent years for many crop plants. In common bean, Blair et al. (2013) reported the first 736 SNP chip and Song et al. (2015) then developed the BARCBean6K_1 BeadChip with > 5000 SNPs that has now been utilized in several genetic studies (Cichy et al. 2015a, b; Kamfwa et al. 2015). Genetic studies have also been carried out employing other SNP genotyping platforms, like KASP genotyping at LGC Genomics service provider (http://www.lgcgroup.com, (Diaz et al. 2018), the Fluidigm platform (www.fluidigm.com) or by Genotyping-by-sequencing (GBS) (Hart and Griffiths 2015).

Marker-assisted selection (MAS) is the major method of molecular breeding, whereby a phenotype is predicted based on the molecular markers results. MAS has been made possible by a multitude of genetic studies that identified the associations between the trait of interest and genetic regions, harboring genes for such traits—like disease resistance (Miklas et al. 2006). SNPs tagged with disease resistance were recently published for Bean Common Mosaic Necrotic Virus (BCMNV, Bello et al. 2014), Anthracnose (Zuiderveen et al. 2016), Fusarium root rot (Hagerty et al. 2015) and Angular Leaf Spot (ALS) (Keller et al. 2015).

The aim of this project was to establish a SNP genotyping platform as a community resource, and develop a set of genotypic data using SNPs as a reference for scientific research of the community. In this study, SNP markers were used to fingerprint a set of 708 released bean varieties, landraces and breeding lines mainly from the Pan African Bean Research Alliance (PABRA) network and the CIAT bean breeding program. Analyses of the data set were carried out: (1) to determine the diversity of the materials used by African breeding programs, (2) to evaluate quality and integrity of lines collected from many programs, (3) to show examples for genetic studies and for tracking of introgressions, and (4) to demonstrate examples of the application in MAS.

## Materials and methods

### Assembly of germplasm

A set of 722 common bean lines were initially assembled at CIAT in Colombia and Uganda, 708 of them were genotyped with high quality data. The entries were collected in five sets (Table 1). The set 1 lines were selected to represent the diversity of the CIAT breeding program including two lines of *Phaseolus acutifolius* A. Gray and *Phaseolus coccineus* L. that had been used in interspecific crosses. Set 2 lines were compiled from African partners in Burundi, Ethiopia, Kenya, Uganda, Tanzania, Malawi, Zambia, Zimbabwe, Rwanda, DRC, RSA, consisting of breeding lines, released varieties and land races. Set 3 included lines sourced from the Andean Diversity Panel as described by Cichy et al. (2015a) that is being maintained at CIAT in Uganda and other African germplasm. Set 4 included breeding lines and parental lines of populations for genetic studies from Zimbabwe and CIAT. Set 5 was composed of released varieties from Zambia (Maredia et al. 2016). In general, the germplasm in this study included released and pre-released varieties developed to address specific constraints in Africa, breeding lines evaluated in several countries, and those routinely used as parental lines (Online Resource 1 provides detailed germplasm information). Seed is available from CIAT, Uganda.

**Table 1:** Overview of germplasm sets genotyped

Name	Project number	No. samples	No. SNPs	Genotyping platform	Germplasm origin
Set 1	CIAT set1	94	1497	LGC KASP	CIAT breeding diversity
Set 2	1583.013-02	94	770	LGC KASP	Several African programs
Set 3	1583.020-02	296	732	LGC KASP	Several African programs
Set 4	1583.030.-02	194	788	LGC KASP	Zimbabwe and CIAT
Set 5	1583.040	24	765	LGC KASP	Zambia

### Leaf sampling and DNA extraction

Seeds of the collected germplasm were germinated under screenhouse conditions at Kawanda research station in Uganda. At 2–3 weeks, leaf samples were collected using a leaf sampling kit provided by LGC (formerly KBioscience, http://www.lgcgroup.com) to facilitate the cutting of leaf discs, transportation and concomitant desiccation for eventual DNA isolation. One kit included one 96-well storage plate, one perforated, gas permeable, heat-sealable film seal, 50 g desiccant pack, two heavy-duty sealable bags, a Harris Uni-Core leaf cutting tool for cutting 6 mm leaf discs and a Harris self-healing cutting mat. To take a sample, a leaf of a specific entry was placed on a cutting mat and a disc cleanly cut using an uncapped cutting tool in a rolling, circular motion. The disc from each of the plants was plunged into a single well of the 96-well storage plate. Once a storage plate was filled to capacity it was covered with the seal film and sealed placing a hot household iron on top of the film seal, ensuring all wells receive a heat treatment of about 2 s. The filled plates and desiccant packs were placed inside the sealable bag, with the majority of the air removed, and shipped to the service provider in UK for DNA isolation.

### Genotyping

The outsourcing genotyping platform at LGC Genomics using KASP™ chemistry was employed, which currently holds > 1500 SNP assays for common bean. SNP markers were established through a cooperation with the BeanCAP project (http://www.beancap.org/) that developed the Illumina BARCBean6K_3 SNP BeadChip (Cichy et al. 2015a; Song et al. 2015). Set 1 was genotyped with ~ 1500 markers. Based on that data set, a binning procedure was carried out to remove SNP groups that have largely the same information content (position and genotyping pattern), leaving ~ 800 markers for the following genotyping runs. In the five genotyping sets, a total of 722 genotypes were evaluated with ~ 800 SNP assays. Raw data is available in Online Resource 2.

### Data analysis

From the 722 samples that were genotyped, 708 samples were analyzed together after filtering for < 50% missing data, high heterozygosity, or samples of unknown identity (Online Resource 2). Samples with the same name that appeared twice in a given genotyping set were marked with extensions “_r1”, “_r2”, etc., indicating the replicates. SNP markers were filtered using only those that had < 20% genotyping errors (marked as “?”) and had generated genotypic data for at least 50% of samples. However, four diagnostic disease markers were retained even though they were evaluated in < 50% of samples. Three of those four SNP markers tag the *bc*-*3* gene for BCMNV resistance (bc3, bc-3a, Bc-3b) and one bruchid resistance (IntRegAPA3). In total, 754 markers were used for the comparative analysis. Genotyping data were visualized and a correlation matrix (Online Resource 3) was generated in Flapjack (Milne et al. 2010). A phylogenetic dendrogram was constructed using 708 genotyping samples and 754 markers, by the NGSEP bioinformatics program to compare SNPs (Duitama et al. 2014) and visualized by SplitsTree4 (Huson and Bryant 2006).

Pairs of identical germplasm showing less than 1% homozygous mismatches, as presented in Online Resource 4, were identified with the NGSEP module to compare Variant Call Format (VCF) files. This module was implemented to compare a VCF file against itself to calculate the number and the percentage of homozygous and heterozygous differences between every pair of samples. The module output provides the number of variants genotyped in each sample of the pair, the number of variants genotyped in both samples, the number of heterozygous differences, percentage of heterozygous differences, the number and percentage of homozygous differences, the number of total differences and the percentage of total differences matrix.

For the introgression mapping, consensus haplotypes for indicated sample pairs were generated by (1) selecting the available genotype call where the other line had a missing data call, (2) selecting a homozygous genotype call in case the other line had a heterozygous genotype call, and (3) dis-selecting markers where both lines showed different homozygous calls or missing data. For visualization of segregation patterns monomorphic markers between parental lines were removed.

## Results

Genetic fingerprinting data were generated on 708 genotypes, collected from 10 African bean programs, a comprehensive representation of released varieties, landraces and breeding lines utilized in Eastern and Southern Africa, as well as diverse breeding lines from the CIAT breeding program in Colombia (Table 1, details on germplasm in Online Resource 1). SNP fingerprinting on lines from African breeding programs was part of the Generation Challenge Programme (www.generationcp.org) activities, a strategy aimed to enable small breeding programs to utilize SNP genotyping tools through outsourced genotyping service. Altogether, five germplasm sets were genotyped with 732–1440 SNP based markers (Table 1), the original genotyping data is available in Online Resource 2.

### Population structure

**Fig. 1:**
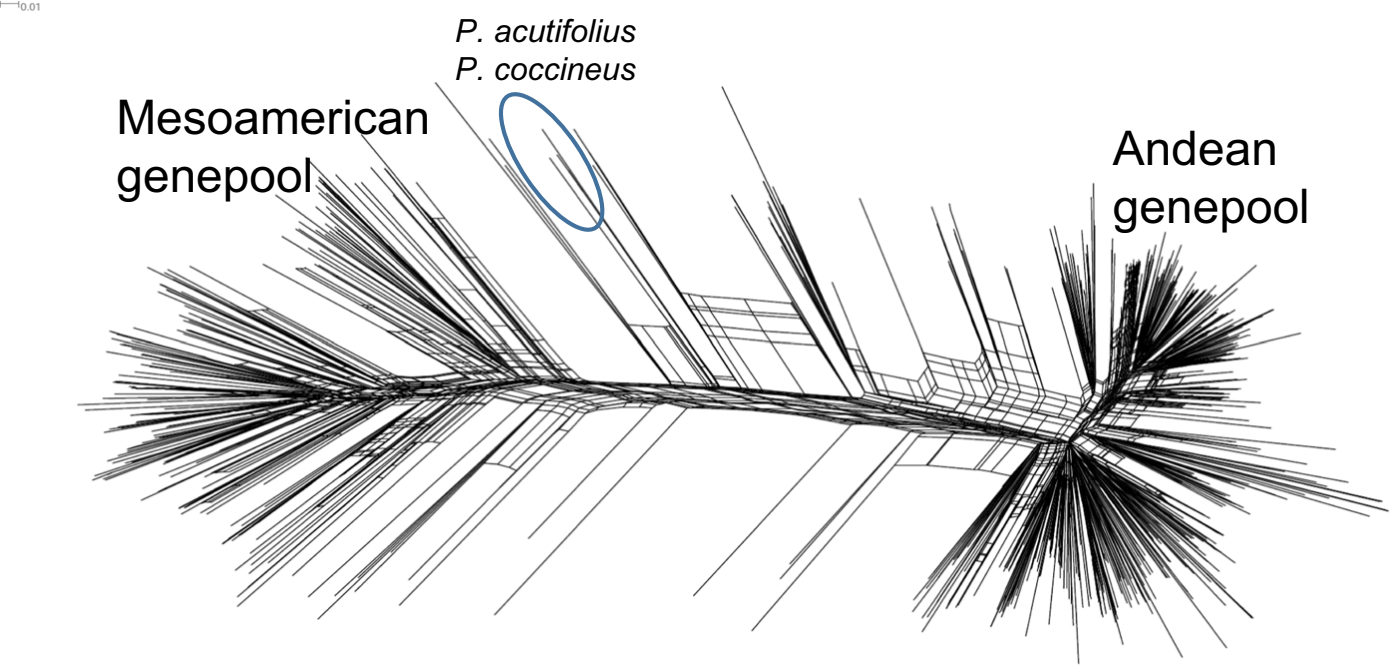
Population structure overview of 708 genotypes. A dendrogram was prepared based on 754 SNP markers, classifying most lines into either the Andean or Mesoamerican genepool. Online Resource 5 provides a file for this dendrogram which allows to zoom and different dendrogram options, to be viewed with SplitsTree4 software (Huson and Bryant 2006)

A dendrogram was constructed of 708 genotypes using 754 high quality markers (Fig. 1 and Online Resource 5 which provides a zoomable higher resolution dendrogram to be viewed with SplitsTree software). The population structure showed clear separation of the Andean and Mesoamerican gene pools, with the majority of lines belonging to the Andean genepool in line with the general preference for large seeded grain types in Eastern and Southern Africa. The dendrogram did not reveal clear clusters that could be associated to races and grain classes, keeping in mind that the information on races of samples is mostly not available.

Detailed information on the relatedness among bean samples are observed in Online Resource 5, which could aid in selection of parental genotypes or other activities. For instance, closer inspection of several branches revealed a number of similar materials, e.g., the CAL96 cluster harboring six genotypes (CAL96_set1, CAL96_ set2, CAL96_set3, K132_set3, Mbereshi_set3, and MAZ216_set4) with no significant genetic variation. Different replicates of CAL96 were expectedly very similar to K132, which is known to be the name of this variety released in Uganda (Online Resource 1). Other lines that appear identical include the Zambian released variety Mbereshi and MAZ216, which reveals an error as this is not expected to be identical based on its pedigree. The issue of multiple names for the same germplasm was a very important finding and will be given more attention below. The tree view also allowed identification of more obvious errors, e.g., placement of ICA_Quimbaya_set3, a known Andean large red seeded genotype, within a Mesoamerican branch, which likely resulted from the mixture of seeds of different varieties.

Interestingly, the two samples of sister species *P. acutifolius* and *P. coccineus* did not group apart as distinctly as would be expected, which is likely attributed to the selection of SNPs (Fig. 1). SNPs were selected based on *P. vulgaris* germplasm, and therefore they do not capture the vast additional variability in sister species.

### Genetic correlation analysis identifies identical germplasm samples

**Fig. 2:**
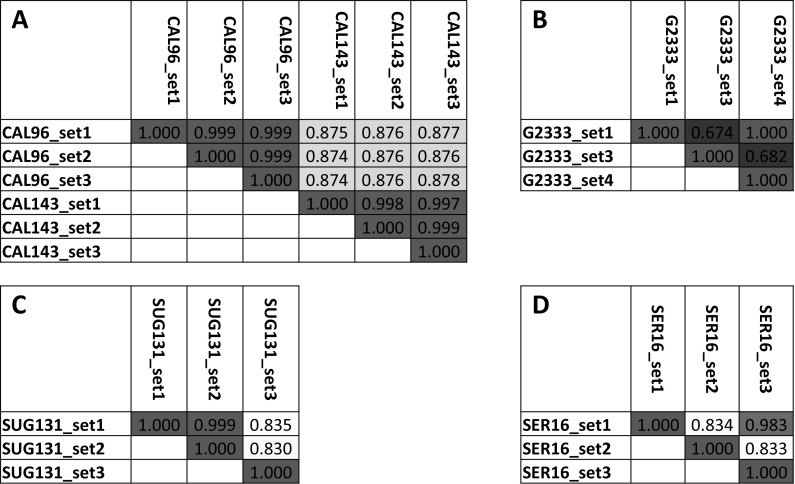
Genotypic similarity of selected genotypes, utilized for quality control of seed sample integrity. **a**–**d** are excerpts of the complete similarity matrix with all genotypes (Online Resource 3), showing pair-wise similarity based on 754 markers. **a** Example of the same genotypes received from different institutes, showing identity of these genotypes (correlation > 0.99). **b**–**d** Examples of genotypes where samples from different origins are not identical revealing probable seed mix-ups. Color code depicts degree of similarity from blue to red. (Color figure online)

**Fig. 3:**
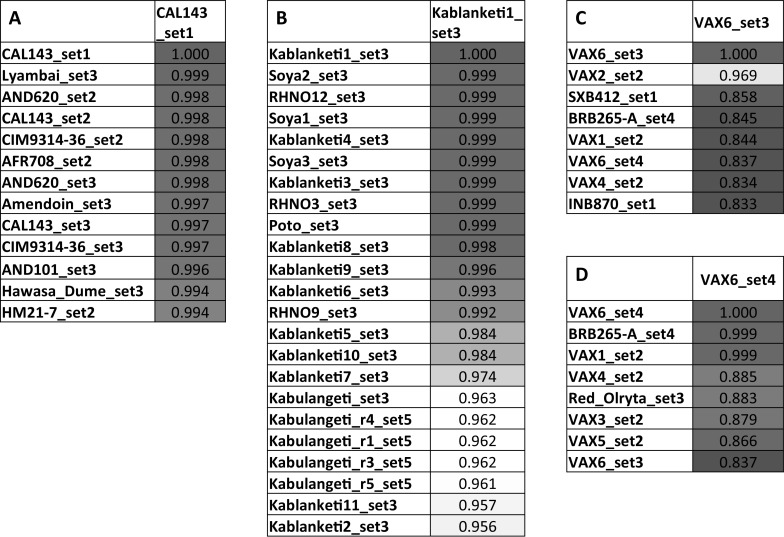
Genotypic similarity of selected genotypes, examples for identification of identical lines. **a**–**d** are excerpts of the complete similarity matrix with all genotypes (Online Resource 3). **a** All genotypes sorted by similarity to CAL143_set1. 12 genotypes have correlation > 0.99 suggesting identical germplasm. **b** 12 genotypes that appear identical to Kablanketi1_set3. **c**, **d** Sorting by similarity to VAX6_set3 shows low similarity to VAX6_ set4. Sorting by similarity to VAX6_set4 reveals its identity to VAX1_set2. Color code depicts degree of similarity from blue to red. (Color figure online)

A complete genetic correlation matrix was created using Flapjack software, displaying pairwise similarities between germplasm samples based on SNPs genotyped in each pair (Online Resource 3, see caption for instructions on searching and sorting). Figures 2 and 3 demonstrate some applications of this data set.

Figure 2 investigates germplasm integrity and quality control, showing correlations of germplasm from different sources that are expected to be identical. Well known varieties CAL96 and CAL143, sent by different institutes, prove to be identical, using a threshold of a pairwise genetic correlation > 0.99 (Fig. 2a). This demonstrates that the methods are well suited to provide the expected results on replicates.

Figure 2b–d show that samples with the same name are not always identical in genotype. Different samples of each G2333 and SUG131 from independent origins were significantly different, indicating mixed seed samples. Mix-ups of this kind are unfortunately common in these data sets, likely due to accumulated errors of seed management from many individuals and institutes over a long time period or during sample handling in the fingerprinting activities. A quantitative estimate of mix-up in this data set is hindered by the issue of duplicated names and often unknown line release history and therefore in many cases it is not clear what samples should or should not be identical.

A number of samples (BRB265-A_set4, G5686_set3, ICA_Quimbaya_set3) that are known to belong to the Andean gene pool, were clustered with Mesoamerican lines. Conversely, there were also several genotypes (Hawasa_Dume_set3, RAZ103_set4, SEQ11-A_set4) known to be Mesoamerican that clustered with the Andean gene pool. Presence of re-selections e.g., BRB265-A and BRB265-B, indicate that the breeder selected different lines out of the same seed batch, usually based on segregating seed types. These are mostly quite different, suggesting seed contaminations rather than residual segregation.

Another mismatch was observed between two samples of the CIAT breeding line, SER16; SER16_set1 and SER16_set3 had a correlation coefficient of 0.982 with 11 homozygous mismatches (Online Resource 3). Even though a correlation of 0.98 indicates high similarity, it did not meet the threshold to be considered identical. This may represent differences originating from residual heterozygocity during line coding, which is often based on F4 or F5 individual selections at CIAT. This example shows that line coding as commonly performed in breeding programs leaves certain ambiguity, which cannot be easily resolved as these sister lines may or may not show different agronomic properties in specific conditions. The authors suggest to continue using a threshold of 0.99 to consider lines identical with an acceptable degree of certainty.

Beyond these examples of re-selections and residual heterozygocity, the data set contains over 30 significantly different sample pairs that share the same code, particularly in germplasm set 4, that cannot be explained other than errors.

Next to unexpected differences between samples, the data set also revealed previously unknown identity between lines. A complete list of pairs of duplicate samples is available in Online Resource 4, identity was determined based on number of homozygous mismatches rather than correlation coefficients, using a threshold level of < 1% homozygous mismatches. Duplicate lines were quite rare with 65% of samples in the data set having no duplicates and only 9.8% having more than two duplicate lines (Online Resource 3 and 4). Twelve genotypes appeared identical to either CAL143_set1 or Kablanketi1_set3, which appear to be very popular genotypes (Fig. 3a, b). This could have resulted from a number of causes such as; several different names being assigned to the same genotype when it was released in different countries, farmers introducing particular names for popular varieties, and/or as a result of seed mix-up. Again, another error was also observed for the a small seeded Mesoamerican variety Hawasa Dume (0.44% homozygous mismatches with CAL143) which should not be mistaken for the large seeded CAL143.

In addition, the analysis of the similarity matrix (Online Resource 3) can help to resolve certain data integrity issues. For example, after sorting all samples by similarity to VAX6_set3, notably VAX6_set4 did not show a high similarity indicating an error (Fig. 3c). Sorting by similarity to VAX6_ set4 resolved this issue (Fig. 3d), revealing that VAX6_set4 is actually identical to VAX1_set2. This most likely implies that a mix-up of seed batches might have occurred between VAX6 and VAX1. These examples demonstrate that the data set is diagnostic to confirm expected duplicates and identify previously unknown identities in germplasm.

### Identifying polymorphic markers between parental lines and tracking of introgressions in offspring

**Fig. 4:**
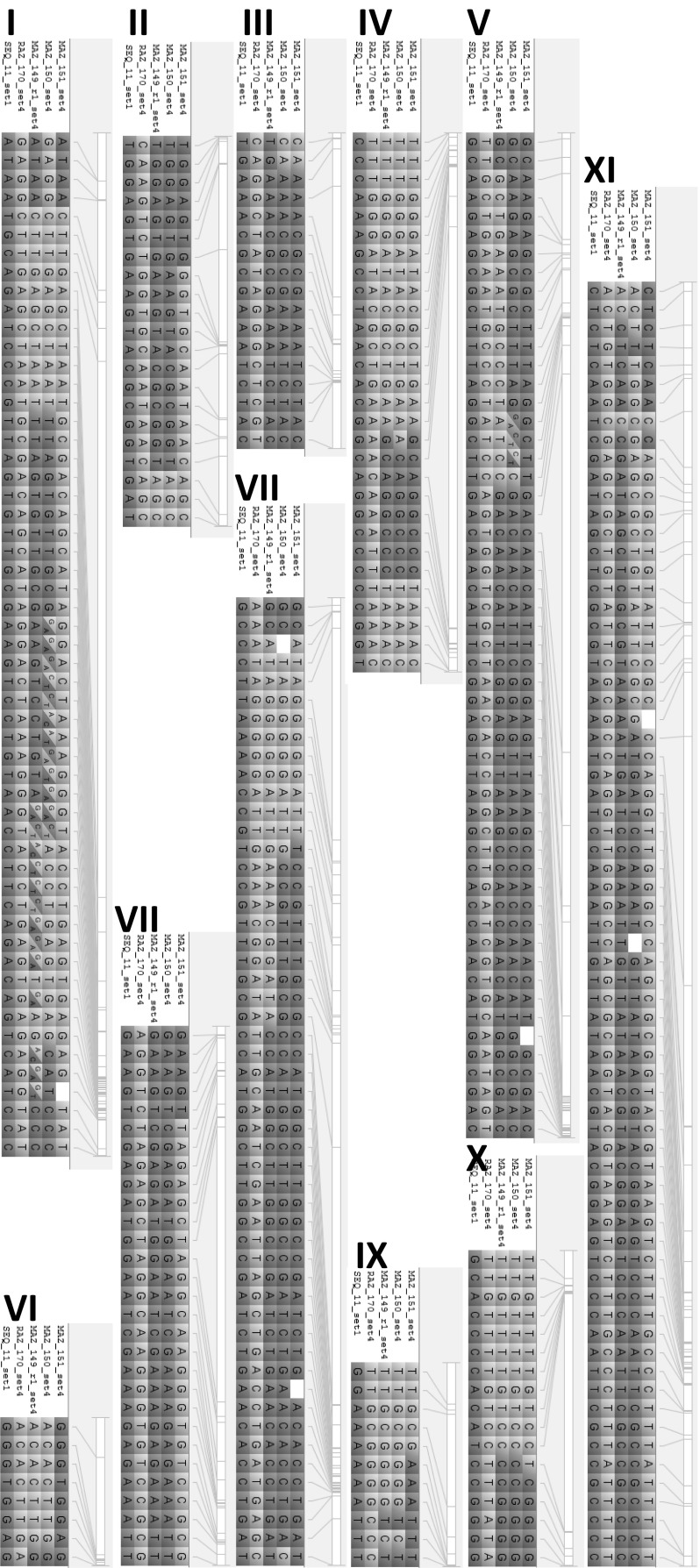
Visualization of segregation patterns in three MAZ lines derived from the cross SEQ11 X RAZ170. Segregation pattern between parental lines SEQ11 and RAZ170 and offspring lines MAZ149, MAZ150 and MAZ151 is shown, using 363 polymorphic markers. RAZ170 alleles in yellow, SEQ11 in blue, physical marker positions are indicated next to each chromosome. (Color figure online)

Fingerprinting data allows to track the segregation of alleles from the parental lines in the resulting offspring lines after generating the cross. This was demonstrated on the three MAZ lines that were derived from the cross SEQ11 × RAZ170 (Fig. 4). The chromosomes are expectedly composed of a mosaic of both parents, forming large blocks of alleles (haplotypes) from either parent displaying between 0 and 4 recombinations per chromosome. These MAZ lines were developed to introgress the resistance to bruchids (*Zabrotes* sp.) originating from the Mesoamerican RAZ lines. Notably, all three MAZ lines have the allele from the resistant donor parent RAZ170 at the end of chromosome 4 (last five markers are positioned between 43.9 and 45.5 MB, Online Resource 2). This region holds the arcelin locus, a major locus reported for bruchid resistance at ~ 44.2 MB (Blair et al. 2010). The MAZ lines likely retained the resistant allele at this locus as a result from their phenotypic selection for bruchid resistance. The marker IntRegAPA3 was developed to tag this locus and additional polymorphic markers can be selected using the fingerprinting data set to monitor the segregation of this locus in crosses with other parents.

The co-dominant nature of SNP markers using KASP assays allows for the identification of heterozygous loci, indicating an advantage over the early generation of dominant markers such as SCAR and RAPD, or high density GBS data which often cannot score heterozygous genotypes unequivocally. Blocks of heterozygous genotype calls were observed e.g., in chromosome 1 for the lines MAZ149 and MAZ150 (Fig. 4), which are indicative of truly heterozygous chromosomal regions, whereas single, interspersed heterozygous calls might be due to genotyping errors. These advanced lines are not expected to have large heterozygosity, and hence the identified regions are likely representing residual heterozygosity that was still segregating after the lines were coded. The identification of heterozygosity can also be useful for breeding purposes to differentiate hybrids from the self-pollinated individuals in F1 generation, as the fingerprinting data set allows to select polymorphic markers between parental lines for such evaluations.

Backcross breeding usually results in transferring small genomic regions from an exotic crossing parent into breeding lines of choice. Identification of these introgressions that may hold valuable alleles can be very useful information for monitoring the breeding process (Ferreira et al. 2016).

**Fig. 5:**
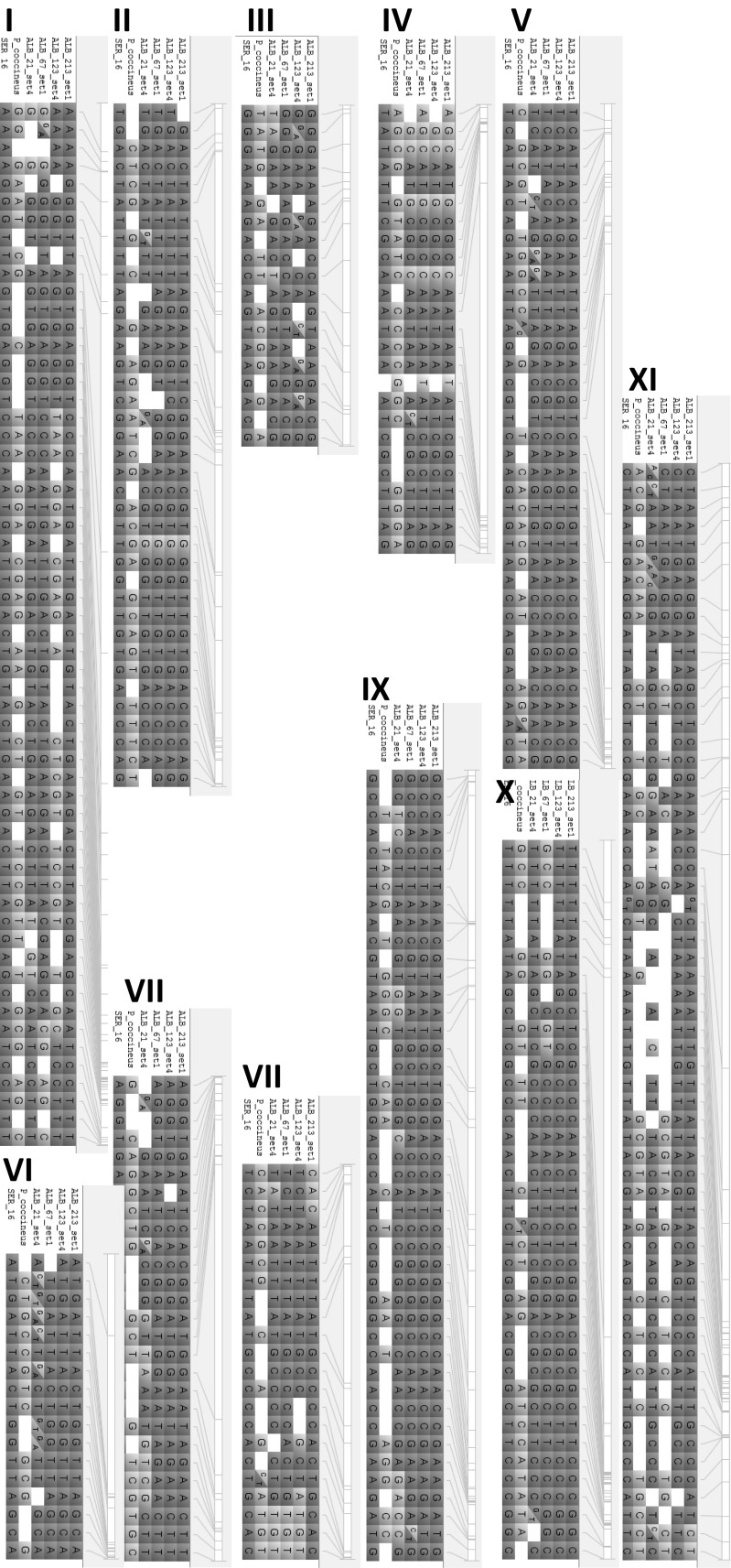
Visualization of segregation patterns in four ALB lines derived from the cross SER16 × (SER16 × G35346). Segregation pattern between parental lines SER16, G35346 (*P. coccineus*) and offspring lines ALB21, 67, 123, 213 is shown, using 388 polymorphic markers. SER16 alleles (a consensus sequence of samples SER16_set1 and SER16_set3) are depicted in blue, G35346 alleles (a consensus of G35346-3Q_set1 and G35375-2P_set1) in yellow. Physical marker positions are indicated next to each chromosome. (Color figure online)

An example of tracking interspecific introgressions of *P coccineus* accession G35346 into breeding line SER16 is visualized in Fig. 5. ALB lines that were derived from the cross SER16 × (SER16 × G35346) show several large introgression blocks from the parent G35346. Sister species have fewer SNP calls due to the significant genome differences, hence, after removing monomorphic and poor quality markers, only a list of 388 polymorphic SNPs were investigated. Of these 388 SNP markers 137 had missing data calls in G35346. Markers with missing data were retained because they can be used as dominant presence/absence markers in this case. An introgression of a large fragment on chromosome 1 (Fig. 5) with the same missing data calls in the ALB lines as well as in the parent G35346 indicates that the whole chromosomal region was successfully transferred to *P. vulgaris*. Introgressions in the four ALB lines ranged from 3.1 to 16.3%, below the ratio of 25% expected from the backcross population SER16 × (SER16 × G35346), which was likely due to selection against genetic drag from exotic alleles associated with poor agronomic performance. In summary, fingerprinting of parental lines and progenies reveals polymorphic markers that can be used for quality control and evaluation, tracking plus visualization of the breeding progress.

### Marker assisted selection using LGC KASP platform

Marker-assisted selection (MAS) is the principal molecular breeding approach by which a phenotype such as disease resistance is predicted based on a molecular marker. To demonstrate the usefulness of this SNP platform for MAS, three markers were genotyped including two SNPs in the *eIF4E* gene that were previously reported to be associated with the *bc*-*3* resistance gene to Bean Common Mosaic Virus (BCMV)/Bean Common Mosaic Necrotic Virus (BCMNV) (Hart and Griffiths 2013; Naderpour et al. 2010). As these markers were established later during the project, not all germplasm sets have been evaluated with these markers. Additional phenotypic data are only available for few genotypes (Table 2). Markers bc3 and bc-3a query the same SNP (C227A), hence, marking the same samples with the resistance associated allele A:A, Bc-3b is based on a different SNP (T194A) in the same *eIF4E* gene. The resistant allele is detected in lines known to harbor BCMV resistance, like the BRB and RCB lines, as well as in lines not previously known to have the *bc*-*3* resistance gene like MCM1015 and RWV1129 for which phenotypic information is not available. Available data for BCMV/BCMNV resistance correlate well with the data from all three markers, which shows that these markers are suitable for MAS. Most African varieties display the susceptible alleles (C:C/T:T). The genotyping using these specific markers demonstrates the usefulness of this SNP platform for MAS. This allows breeders to use SNP markers in an easy and cost-effective way without having a molecular analysis infrastructure.

**Table 2:**
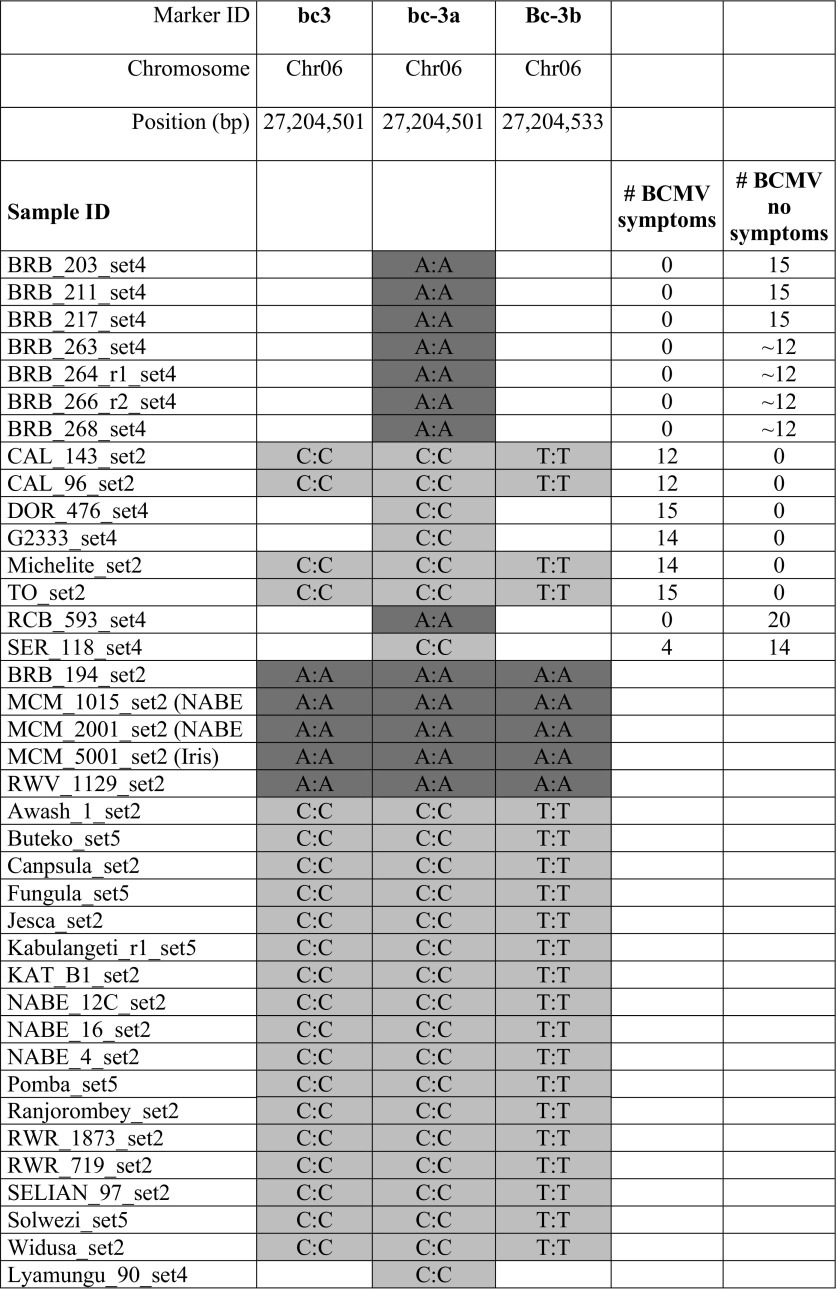
Marker assisted selection for BCMV/BCMNV with *bc*-*3* markers

Three markers were designed for two SNPs in the *eIF4E* gene of the *bc*-*3* locus, providing resistance to BCMV/BCMNV. Selected genotypes are displayed for which phenotypic and/or genotypic data is available. Phenotypic evaluation counts number of plants with or without symptoms. Genotypic data is an excerpt from full data set in Online Resource 2

## Discussion

This study presents a new genetic tool for the bean community including a SNP genotyping platform, a large data set and a number of practical applications. This tool is intended for African bean improvement programs, as germplasm sets were selected from breeding materials and the outsourcing genotyping platform may be used by programs that don’t have the facilities for in-house SNP genotyping.

This platform has potential to be used for: (1) the identification and conservation of new and unique germplasm to protect agrobiodiversity; (2) Common bean improvement through parental line selection and MAS; (3) Support of seed dissemination projects and official variety release systems in variety cataloguing; (4) Estimation of the extent of national and regional variety spread, use and adoption of improved varieties and regional trade; (5) Identification of varieties to ensure seed purity.

### Characterization of germplasm diversity

Analysis of germplasm diversity shows that both Mesoamerican and Andean gene pools are in use, with an emphasis on large seeded Andean genotypes, which represents the known preferences of Eastern and Southern African farmers and consumers. Whereas the genepools are very distinct, the phylogenetic dendrogram does not result in clear separation of the races or regions of origin as reported by Cichy et al. (2015a) where North American grain types form distinct clusters while South American and African lines mostly group together, or by Lobaton et al. (2018) clustering separately the races Durango and Mesoamerica. African germplasm originated from different regions including North- and South American breeding efforts, hence, no clear distinction is expected. Also, race affiliation or admixture is not known for most lines, which makes it difficult to identify these from the dendrogram.

Knowledge on population structure can assist breeders in parental line selection. Crossing between closely related lines should be avoided to have enough genetic variability for effective selection. Also crosses between gene pools are avoided by many breeders as it is difficult to regain commercial grain types and agronomic performance. The available data set may aid breeders in selection of crossing parents for optimal genetic gain. In particular, the identification of duplicates or identical lines through different means is important to avoid a waste of resources.

Evaluation of sister species *P. acutifolius* and *P. coccineus* samples reveals limitations of this SNP set. Genotyping calls are significantly lower with missing data rates of 23–41%, suggesting that SNP assays fail due to sequence deviation. The SNPs employed in the study are not able to properly group apart the sister species (Fig. 1) compared to other methods like AFLPs (Muñoz et al. 2006), chloroplast polymorphisms (Desiderio et al. 2012; Chacón et al. 2007), or whole genome re-sequencing data (Rendón-Anaya et al. 2017; Lobaton et al. 2018). Because SNPs were selected within *P. vulgaris*, the stark genetic variability of sister species is not revealed. This exemplifies the intrinsic problem of pre-selected SNP panels like chips or the LGC KASP platform, which can only detect variation as designed for. While it is well suited to evaluate the diversity of breeding materials from both Andean and Mesoamerican gene pools, the exotic or interspecific variation may not be captured.

### Quality control and monitoring of seed sample integrity

This platform can be a useful tool to evaluate the identity and purity of breeding lines. While many samples that were entered in several replicates or by several institutes were found to be identical based on a similarity threshold of > 0.99 (Fig. 4), it has to be stated that a significant number were not. Overall, this data set reveals a surprising and worrying number of inconsistencies.

Some samples belonging to the Andean gene pool based on their IDs cluster with Mesoamerican lines and vice versa. There are a number of re-selections (e.g., BRB265-A and BRB265-B) that are mostly quite different, suggesting seed contaminations. Another issue is that some sample pairs like SER16_set1 and SER16_set3 are not completely identical, likely due to residual heterozygosity at line coding stage, a common issue in breeding programs. In addition, over 30 significantly different sample pairs that share the same code cannot be explained other than errors. Germplasm has been managed by many institutes with changing staff, at times over decades. Errors may occur at many stages, during shipments, sowing, local multiplication, seed storage, recoding and also at germination, DNA extraction and sample handling during this project. It is important to identify these cases and not to rely completely on germplasm identifiers.

Next to inconsistencies between entries with identical names, analyses revealed groups of identical germplasm with different names. These are partially explained by renaming popular varieties during releases in different countries or through unofficial germplasm exchange. This is important not only for crosses but also for dissemination of new varieties in order to avoid investments in promoting identical germplasm. The fingerprinting method has been applied in an adoption study for common bean in Zambia with the purpose of identifying released varieties in samples collected from farmers (Maredia et al. 2016). Application of genetic fingerprinting methods is an invaluable tool for correct data interpretation and unbiased estimates of impacts of varietal adoption.

In addition, some confusion can arise from germplasm named after grain types, like Kablanketi, White or Yellow. These do not represent varieties, but groups of varieties with the same grain type. There were 18 Kablanketi samples in this data set, most are quite similar (apart from Kablanketi Ndefu), but genetically they formed 5–6 groups of germplasm.

These issues listed here on inconsistencies in collected germplasm and multiple germplasm naming are very important findings. These data exemplify the need for an easy to use genotyping platform to make use of DNA fingerprinting for quality control in breeding, trials, dissemination and germplasm conservation.

Data generated from SNP fingerprinting are very important for quality control and quality assurance (QC/QA). Major breeding companies have been using such resources for QC/QA very successfully for some time. Some are of the opinion that QC/QA applications have been the major impacting utility of SNP technologies. For this reason, KASP based QC/QA SNP panels are being developed in the Excellence in Breeding project (http://excellenceinbreeding.org), that can be applied with the SNP platform described here.

### Germplasm nomenclature

A further issue is the lack of a gold standard in nomenclature. Apart from several spelling errors, sample names were frequently spelled in different versions, such as Kabulangeti vs Kablanketi, Ranjonomby vs Ranjorombey, Lyamungo vs Lyanmungu, Red Wolaita vs Red Wolayta and Jesca vs Hesca, only to name a few. Also abbreviations are non-standardized, such as RED CANADIAN WONDER and R.C.Wonder, or MDRK and Michigan Dark Kidney Red probably originate from the same term. On top of that spacing and capitalizations appear at random.

We suggest nomenclature rules in this work: Line codes and numbers are not separated (e.g., SEQ1), only names are separated by one space for readability (e.g. Natal Sugar). Re-selections within a line are indicated by hyphenated numbers (MD23–24). Line codes and institutions/abbreviations are capitalized (SEQ1) whereas other names only start with a capital letter (ICA Quimbaya). For bioinformatic analyses, spaces and dashes are replaced by underscores if required by the software. The bean community should discuss on setting a standard for nomenclature of genotypes to enable future collaborations in data bases. A data base of germplasm IDs should be created to define and share correct spelling.

### Genetic studies and marker assisted selection (MAS)

This SNP platform has been used successfully for several purposes, like QTL analyses (Diaz et al. 2018), farmer varietal adoption studies (Maredia et al. 2016), or ongoing Marker assisted recurrent selection (MARS) and MAS applications. Here we confirm its usefulness for MAS with markers tagging the known BCMV resistance gene *bc*-*3* (Hart and Griffiths 2013; Naderpour et al. 2010). Next to positive MAS these could also easily be used for negative background selection in introgression programs.

In addition, here we show full visualization of introgressions in intra- and inter specific crosses. This can be used to identify and track introgressions in backcross programs. Genome wide identification of introgressions has also been shown with a higher marker density by (Ferreira et al. 2016). GBS method is more powerful, but requires the capacity for bioinformatics analysis. The SNP platform presented here can furthermore identify heterozygous and segregating genomic regions which can be useful in introgression breeding. This is an advantage over previous low throughput dominant SCAR/RAPD marker systems or high throughput GBS platform which have trouble scoring heterozygocity. Identification of heterozygocity is commonly utilized in breeding to differentiate F1 hybrids from autopolonizations to increase efficiency of breeding programs.

The study demonstrated that this platform can be effectively used for MAS for disease resistance, to identify heterozygotes to confirm F1 of hybridizations, and quality control. The SNP platform holds a number of advantages over other genotyping methods. SNPs can be selected from a large pool to fit each experiment, e.g., polymorphic and well-spaced SNPs for specific populations (Diaz et al. 2018). Also any number of germplasm entries can be tested and no specialized bioinformatics expertise is required. Other genotyping methods like chips, GBS or fluidigm are more rigid in format requirements (specific number of SNPs and samples) to work efficiently. This genotyping platform is characterized by a high degree of flexibility and cost effectiveness per data point for SNP analyses in small to medium scale, from a few SNPs up to 200 or more SNPs. In addition to the convenience of sending leaf materials and outsourcing DNA extraction and SNP genotyping, this platform can be considered as an effective molecular breeding tool for breeders that don’t have access to SNP genotyping infrastructure. Together with the available genotyping data and methods, this new resource can significantly impact African breeding programs.

## Electronic supplementary material

Below is the link to the electronic supplementary material.

Online Resource 1 Information on evaluated germplasm. Available data on all lines investigated in this study, mostly provided by partners that shared germplasm (XLSX 71 kb)

Online Resource 2 Genotyping data, raw and analyzed. Sheet overview: Sheet set 1 CIAT: Raw data of data set 1, called CIAT 1st plate, Sheet set 2 (1583.013-02): Raw data of data set 2, called 1583.013-02, Sheet set 3 (1583.020-02): Raw data of data set 3, called 1583.020-02, Sheet set 4 (1583.030.-02): Raw data of data set 4, called 1583.030.-02, Sheet set 5 (1583.040): Raw data of data set 5, called 1583.040. Sheet combined: Data of all 5 data sets combined, with additional information and filtered for bad genotypes and markers. Heterozygous and missing data calls indicated by colored cells. Sheet FlpJk_genot: A text file of this sheet can serve as one of the two input files required for FlapJack software. Sheet FlpJk_map: A text file of this sheet can serve as one of the two input files required for FlapJack software (XLSX 7302 kb)

Online Resource 3 Similarity matrix showing the correlations between all analyzed genotypes, for searching and sorting. Sheet: a SimilarityMatrix. Matrix of pairwise genetic similarity between all 708 evaluated lines, using 754 SNP markers. row “# of samples > 0.99 similar” indicates how many further samples are identical to the genotype stated above. Sorting (in Excel 2013): to sort the genotypes in order of similarity to a certain sample do the following: select a column by clicking on a letter above the cells, e.g., “B” above “A222_set1” click the “data” tab, click “Sort ZtoA” accept “expand selection”. This sorts all rows of genotypes, to start with the one most similar to “A222_set1”, revealing similar or identical genotypes. sheet: b matches and mismatches. A matrix with pairwise comparisons of the number of matched, and mismatched genotyping calls between any pair of samples. For any sample pair this table states the number of identical homozygous genotype calls “ + ”, the number of different homozygous genotype calls “-”, the number of heterozygous genotype calls “het” for each of the two lines, separated by a dash “-”, and the number of missing data points “?” for each line separated by a dash “-” (XLSX 8042 kb)

Online Resource 4 List of identical germplasm samples. All genotype pairs are listed that are considered identical, based on less than 1% homozygous mismatches (XLSX 40 kb)

Online Resource 5 Dendrogram file of all 708 genotypes, corresponding to Fig. 1. The file can be visualized e.g., with SplitsTree4 software (Huson and Bryant 2006), to allow a detailed view on the population structure (TXT 543 kb)
